# Supplementary material for: Nationwide Trends and Projections of Early Onset Gastrointestinal Cancers in China
Source: Cancers (Basel). 2025 Sep 9;17(18):2954. doi: 10.3390/cancers17182954 (PMC12468148; doi:10.3390/cancers17182954)
Supplement: Supplementary file 1 [file cancers-17-02954-s001.zip › Supplementary Table S1.pdf]

Supplementary Table S1. Segment-specific AAPC analysis of incidence and death rate of early-onset gastrointestinal cancers at different year intervals in China

AAPC of incidence rate in esophageal cancer		
Year interval	APC (95% CI)	P-value
1990-1994	1.437 (0.870 - 2.008)	＜0.001
1994-2001	-0.032 (-0.366 - 0.304)	0.853
2001-2007	-4.665 (-5.069 - -4.260)	＜0.001
2007-2010	0.953 (-0.305 - 2.227)	0.138
2010-2016	-2.746 (-3.157 - -2.333)	＜0.001
2016-2021	1.042 (0.477 - 1.610)	＜0.001
AAPC of death rate in esophageal cancer		
1990-1995	1.099 (0.607 - 1.594)	＜0.001
1995-2001	-0.464 (-0.949 - 0.023)	0.0620
2001-2007	-5.932 (-6.390 - -5.471)	＜0.001
2007-2010	-0.017 (-1.451 - 1.439)	0.982
2010-2016	-3.608 (-4.077 - -3.135)	＜0.001
2016-2021	0.065 (-0.579 - 0.714)	0.843
AAPC of incidence rate in stomach cancer		
1990-2002	0.176 (0.082 - 0.269)	＜0.001
2002-2006	-3.363 (-3.907 - -2.815)	＜0.001
2006-2010	1.054 (0.485 - 1.627)	＜0.001
2010-2015	-1.695 (-2.087 - -1.301)	＜0.001
2015-2021	-0.391 (-0.691 - -0.089)	0.0111
AAPC of death rate in stomach cancer		
1990-2002	-0.592 (-0.704 - -0.479)	＜0.001
2002-2006	-5.540 (-6.181 - -4.894)	＜0.001
2006-2010	-0.266 (-0.944 - 0.416)	0.443
2010-2016	-2.899 (-3.252 - -2.544)	＜0.001
2016-2021	-1.730 (-2.202 - -1.255)	＜0.001
AAPC of incidence rate in liver cancer		
1990-1997	3.253 (2.966 - 3.541)	＜0.001
1997-2000	1.469 (0.184 - 2.770)	0.0249
2000-2005	-6.792 (-7.322 - -6.260)	＜0.001
2005-2010	3.879 (3.289 - 4.473)	＜0.001
2010-2014	2.626 (1.802 - 3.456)	＜0.001
2014-2021	-0.107 (-0.447 - 0.234)	0.537
AAPC of death rate in liver cancer		
1990-1999	3.060 (2.685 - 3.437)	0
1999-2004	-7.678 (-8.641 - -6.705)	0
2004-2015	1.779 (1.458 - 2.101)	0
2015-2021	-1.143 (-1.924 - -0.356)	0.005
AAPC of incidence rate in colon and rectum cancer		

1990-1995	2.197 (1.864 - 2.532)	＜0.001
1995-2006	1.124 (0.992 - 1.256)	＜0.001
2006-2010	5.964 (5.319 - 6.614)	＜0.001
2010-2021	3.515 (3.380 - 3.650)	＜0.001
AAPC of death rate in colon and rectum cancer		
1990-1994	0.758 (0.350 - 1.168)	＜0.001
1994-2001	-0.425 (-0.666 - -0.183)	＜0.001
2001-2007	-2.538 (-2.837 - -2.238)	0
2007-2009	4.063 (2.190 - 5.970)	＜0.001
2009-2021	1.011 (0.903 - 1.120)	0
AAPC of incidence rate in gallbladder and biliary tract cancer		
1990-1994	1.682 (1.242 - 2.123)	＜0.001
1994-1998	0.878 (0.262 - 1.498)	0.005
1998-2006	2.310 (2.094 - 2.526)	＜0.001
2006-2010	4.486 (3.848 - 5.128)	＜0.001
2010-2014	2.385 (1.760 - 3.014)	＜0.001
2014-2021	1.550 (1.287 - 1.813)	＜0.001
AAPC of death rate in gallbladder and biliary tract cancer		
1990-1994	1.093 (0.776 - 1.410)	＜0.001
1994-2001	0.456 (0.268 - 0.645)	＜0.001
2001-2003	2.399 (0.973 - 3.846)	＜0.001
2003-2006	-1.971 (-2.656 - -1.281)	＜0.001
2006-2010	2.655 (2.201 - 3.112)	＜0.001
2010-2021	-0.510 (-0.604 - -0.415)	＜0.001
AAPC of incidence rate in pancreatic cancer		
1990-2000	2.002 (1.901 - 2.102)	＜0.001
2000-2006	-0.378 (-0.624 - -0.131)	0.003
2006-2010	4.847 (4.364 - 5.333)	＜0.001
2010-2021	1.239 (1.139 - 1.338)	＜0.001
AAPC of death rate in pancreatic cancer		
1990-2000	1.930 (1.825 - 2.036)	＜0.001
2000-2006	-0.666 (-0.923 - -0.409)	＜0.001
2006-2010	4.738 (4.232 - 5.246)	＜0.001
2010-2021	1.009 (0.905 - 1.113)	＜0.001
