# Supplementary figures and images for: Nationwide Trends and Projections of Early Onset Gastrointestinal Cancers in China
Source: Cancers (Basel). 2025 Sep 9;17(18):2954. doi: 10.3390/cancers17182954 (PMC12468148; doi:10.3390/cancers17182954)

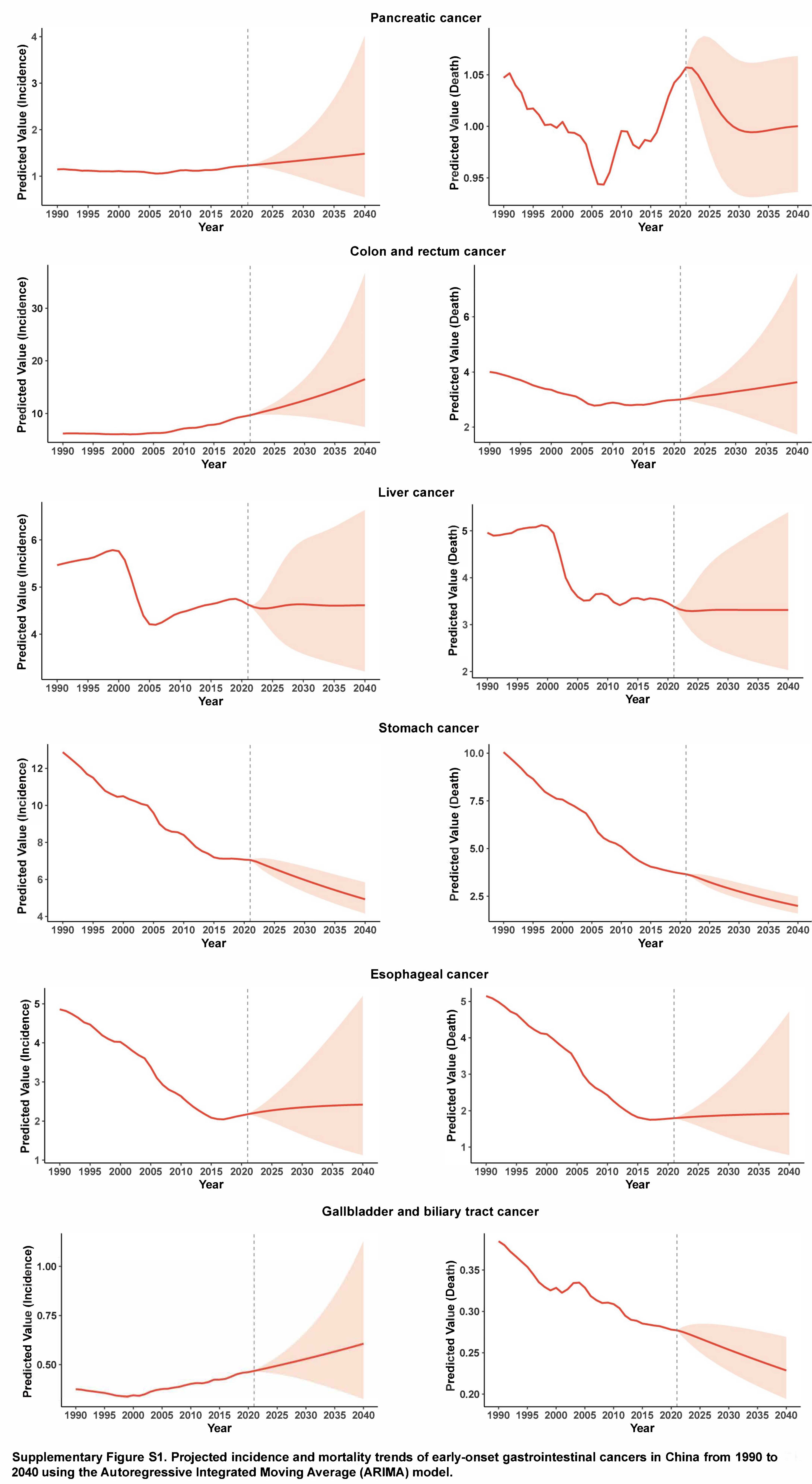

Supplement: Supplementary file 1 [file cancers-17-02954-s001.zip › Supplementary Figure S1.jpg]
