# Supplementary material for: Nationwide Trends and Projections of Early Onset Gastrointestinal Cancers in China
Source: Cancers (Basel). 2025 Sep 9;17(18):2954. doi: 10.3390/cancers17182954 (PMC12468148; doi:10.3390/cancers17182954)
Supplement: Supplementary file 1 [file cancers-17-02954-s001.zip › Supplementary File S1.zip!/Deaths-BAPC_params_output_selected/cause_Stomach_cancer/China_Stomach_cancer_Both_Deaths_BAPC_parameters_and_ppc.docx]

# 1. Methods (BAPC specification)

BAPC with Poisson likelihood (log link) and population offset. Age/cohort: RW2; period: RW1; IID over-dispersion included. Precision priors: log-Gamma(1, 5e-5) for age/period/cohort; log-Gamma(1, 5e-3) for over-dispersion. Intercept: vague Gaussian prior (package default). Training: 1990–2021; projections to 2040 with retrospective prediction enabled. Age-standardization: WHO 2000–2025 weights.

## 1.1. Priors and Hyperparameters

| component | model | prior | param_a | param_b |
| --- | --- | --- | --- | --- |
| age | RW2 | log-Gamma | 1 | 0.00005 |
| period | RW1 | log-Gamma | 1 | 0.00005 |
| cohort | RW2 | log-Gamma | 1 | 0.00005 |
| overdispersion | IID | log-Gamma | 1 | 0.00500 |

## 1.2. Posterior Predictive Coverage

| target | coverage_95 |
| --- | --- |
| ASR (in-sample) | 0 |
| Age-specific counts (in-sample) | 100 |

## 1.3. Residuals – Overall Summary

| stat | value |
| --- | --- |
| mean_resid | -0.02002633 |
| sd_resid | 0.47778587 |
| prop_abs_resid_gt_2 | 0.00000000 |
| acf_lag1 | 0.17918105 |

## 1.4. Residuals – By Year

| Time | mean_pearson | sd_pearson |
| --- | --- | --- |
| 1,990 | 0.083220107 | 0.66198342 |
| 1,991 | 0.105701987 | 0.67112904 |
| 1,992 | 0.126250794 | 0.66209857 |
| 1,993 | 0.115820872 | 0.63733161 |
| 1,994 | 0.104860417 | 0.56590466 |
| 1,995 | 0.124798106 | 0.49856098 |
| 1,996 | 0.095519643 | 0.43301875 |
| 1,997 | 0.041699241 | 0.33496391 |
| 1,998 | 0.019728890 | 0.23017402 |
| 1,999 | 0.006108418 | 0.13974342 |
| 2,000 | 0.039376754 | 0.08530503 |
| 2,001 | -0.029175933 | 0.12364610 |
| 2,002 | -0.067958220 | 0.19846843 |
| 2,003 | -0.011543073 | 0.25397770 |
| 2,004 | 0.033587957 | 0.25688115 |
| 2,005 | -0.086059295 | 0.31572812 |
| 2,006 | -0.330102585 | 0.50793655 |
| 2,007 | -0.348232618 | 0.52908042 |
| 2,008 | -0.240592338 | 0.40345308 |
| 2,009 | -0.064283426 | 0.57897166 |
| 2,010 | -0.018261664 | 0.64904369 |
| 2,011 | -0.112230443 | 0.64931294 |
| 2,012 | -0.263320344 | 0.68179843 |
| 2,013 | -0.238605004 | 0.66521077 |
| 2,014 | -0.122622463 | 0.52045837 |
| 2,015 | -0.134552769 | 0.44286390 |
| 2,016 | -0.070986971 | 0.44846333 |
| 2,017 | 0.018615847 | 0.44077560 |
| 2,018 | 0.090936328 | 0.47226907 |
| 2,019 | 0.106049294 | 0.49292505 |
| 2,020 | 0.151054979 | 0.54126472 |
| 2,021 | 0.234355091 | 0.58811165 |
