# Supplementary material for: Nationwide Trends and Projections of Early Onset Gastrointestinal Cancers in China
Source: Cancers (Basel). 2025 Sep 9;17(18):2954. doi: 10.3390/cancers17182954 (PMC12468148; doi:10.3390/cancers17182954)
Supplement: Supplementary file 1 [file cancers-17-02954-s001.zip › Supplementary File S1.zip!/Deaths-BAPC_params_output_selected/cause_Colon_and_rectum_cancer/China_Colon_and_rectum_cancer_Both_Deaths_BAPC_parameters_and_ppc.docx]

# 1. Methods (BAPC specification)

BAPC with Poisson likelihood (log link) and population offset. Age/cohort: RW2; period: RW1; IID over-dispersion included. Precision priors: log-Gamma(1, 5e-5) for age/period/cohort; log-Gamma(1, 5e-3) for over-dispersion. Intercept: vague Gaussian prior (package default). Training: 1990–2021; projections to 2040 with retrospective prediction enabled. Age-standardization: WHO 2000–2025 weights.

## 1.1. Priors and Hyperparameters

| component | model | prior | param_a | param_b |
| --- | --- | --- | --- | --- |
| age | RW2 | log-Gamma | 1 | 0.00005 |
| period | RW1 | log-Gamma | 1 | 0.00005 |
| cohort | RW2 | log-Gamma | 1 | 0.00005 |
| overdispersion | IID | log-Gamma | 1 | 0.00500 |

## 1.2. Posterior Predictive Coverage

| target | coverage_95 |
| --- | --- |
| ASR (in-sample) | 0 |
| Age-specific counts (in-sample) | 100 |

## 1.3. Residuals – Overall Summary

| stat | value |
| --- | --- |
| mean_resid | -0.02443949 |
| sd_resid | 0.46969159 |
| prop_abs_resid_gt_2 | 0.00000000 |
| acf_lag1 | 0.27348936 |

## 1.4. Residuals – By Year

| Time | mean_pearson | sd_pearson |
| --- | --- | --- |
| 1,990 | 0.06189978 | 0.55958821 |
| 1,991 | 0.09062479 | 0.57181682 |
| 1,992 | 0.08493765 | 0.58511631 |
| 1,993 | 0.08686099 | 0.58697801 |
| 1,994 | 0.07793471 | 0.53007651 |
| 1,995 | 0.08344194 | 0.50716992 |
| 1,996 | 0.08247398 | 0.46906151 |
| 1,997 | 0.06739424 | 0.41881456 |
| 1,998 | 0.06601111 | 0.35319427 |
| 1,999 | 0.06166157 | 0.30214845 |
| 2,000 | 0.09317754 | 0.22994572 |
| 2,001 | 0.02690623 | 0.13667421 |
| 2,002 | -0.03482265 | 0.09644273 |
| 2,003 | -0.02140570 | 0.25820576 |
| 2,004 | -0.03215348 | 0.31828211 |
| 2,005 | -0.14629998 | 0.39166547 |
| 2,006 | -0.34357979 | 0.57708100 |
| 2,007 | -0.36991852 | 0.60890961 |
| 2,008 | -0.30367859 | 0.54643760 |
| 2,009 | -0.18136501 | 0.61121983 |
| 2,010 | -0.12652401 | 0.63349674 |
| 2,011 | -0.17158980 | 0.59309275 |
| 2,012 | -0.23717644 | 0.58516866 |
| 2,013 | -0.20684857 | 0.53790878 |
| 2,014 | -0.07421430 | 0.39470798 |
| 2,015 | -0.06624798 | 0.32974200 |
| 2,016 | -0.04196811 | 0.36541765 |
| 2,017 | 0.03450635 | 0.39718002 |
| 2,018 | 0.11272380 | 0.45761403 |
| 2,019 | 0.13913235 | 0.50169272 |
| 2,020 | 0.16685904 | 0.54753982 |
| 2,021 | 0.23918312 | 0.62715925 |
