# Supplementary material for: Nationwide Trends and Projections of Early Onset Gastrointestinal Cancers in China
Source: Cancers (Basel). 2025 Sep 9;17(18):2954. doi: 10.3390/cancers17182954 (PMC12468148; doi:10.3390/cancers17182954)
Supplement: Supplementary file 1 [file cancers-17-02954-s001.zip › Supplementary File S1.zip!/Deaths-BAPC_params_output_selected/cause_Gallbladder_and_biliary_tract_cancer/China_Gallbladder_and_biliary_tract_cancer_Both_Deaths_BAPC_parameters_and_ppc.docx]

# 1. Methods (BAPC specification)

BAPC with Poisson likelihood (log link) and population offset. Age/cohort: RW2; period: RW1; IID over-dispersion included. Precision priors: log-Gamma(1, 5e-5) for age/period/cohort; log-Gamma(1, 5e-3) for over-dispersion. Intercept: vague Gaussian prior (package default). Training: 1990–2021; projections to 2040 with retrospective prediction enabled. Age-standardization: WHO 2000–2025 weights.

## 1.1. Priors and Hyperparameters

| component | model | prior | param_a | param_b |
| --- | --- | --- | --- | --- |
| age | RW2 | log-Gamma | 1 | 0.00005 |
| period | RW1 | log-Gamma | 1 | 0.00005 |
| cohort | RW2 | log-Gamma | 1 | 0.00005 |
| overdispersion | IID | log-Gamma | 1 | 0.00500 |

## 1.2. Posterior Predictive Coverage

| target | coverage_95 |
| --- | --- |
| ASR (in-sample) | 0 |
| Age-specific counts (in-sample) | 100 |

## 1.3. Residuals – Overall Summary

| stat | value |
| --- | --- |
| mean_resid | -0.003548181 |
| sd_resid | 0.484581706 |
| prop_abs_resid_gt_2 | 0.000000000 |
| acf_lag1 | -0.140154270 |

## 1.4. Residuals – By Year

| Time | mean_pearson | sd_pearson |
| --- | --- | --- |
| 1,990 | 0.277040903 | 0.2772157 |
| 1,991 | 0.287127079 | 0.2719147 |
| 1,992 | 0.157220753 | 0.3266646 |
| 1,993 | 0.112333884 | 0.4021798 |
| 1,994 | 0.081107592 | 0.4417340 |
| 1,995 | -0.024902063 | 0.4845042 |
| 1,996 | -0.108878697 | 0.4941193 |
| 1,997 | -0.233726149 | 0.4951515 |
| 1,998 | -0.273826569 | 0.3649259 |
| 1,999 | -0.291330325 | 0.3500875 |
| 2,000 | -0.036866739 | 0.2913582 |
| 2,001 | -0.192881154 | 0.3273657 |
| 2,002 | -0.033712564 | 0.2850881 |
| 2,003 | 0.348803343 | 0.7275053 |
| 2,004 | 0.412998293 | 0.7702982 |
| 2,005 | 0.170325953 | 0.5179039 |
| 2,006 | -0.128169587 | 0.3501424 |
| 2,007 | -0.171105406 | 0.6687284 |
| 2,008 | -0.185103083 | 0.3787472 |
| 2,009 | 0.001778642 | 0.4514288 |
| 2,010 | 0.081376361 | 0.5792876 |
| 2,011 | -0.014815179 | 0.6781185 |
| 2,012 | -0.241999210 | 0.7290640 |
| 2,013 | -0.309710474 | 0.6436137 |
| 2,014 | -0.095887706 | 0.3732459 |
| 2,015 | -0.114410359 | 0.4540312 |
| 2,016 | -0.034327376 | 0.4524740 |
| 2,017 | 0.048845454 | 0.4520341 |
| 2,018 | 0.134031994 | 0.5918818 |
| 2,019 | 0.089200443 | 0.5062300 |
| 2,020 | 0.074120659 | 0.4042242 |
| 2,021 | 0.101799482 | 0.4577858 |
