# Supplementary material for: Nationwide Trends and Projections of Early Onset Gastrointestinal Cancers in China
Source: Cancers (Basel). 2025 Sep 9;17(18):2954. doi: 10.3390/cancers17182954 (PMC12468148; doi:10.3390/cancers17182954)
Supplement: Supplementary file 1 [file cancers-17-02954-s001.zip › Supplementary File S1.zip!/Deaths-BAPC_params_output_selected/cause_Pancreatic_cancer/China_Pancreatic_cancer_Both_Deaths_BAPC_parameters_and_ppc.docx]

# 1. Methods (BAPC specification)

BAPC with Poisson likelihood (log link) and population offset. Age/cohort: RW2; period: RW1; IID over-dispersion included. Precision priors: log-Gamma(1, 5e-5) for age/period/cohort; log-Gamma(1, 5e-3) for over-dispersion. Intercept: vague Gaussian prior (package default). Training: 1990–2021; projections to 2040 with retrospective prediction enabled. Age-standardization: WHO 2000–2025 weights.

## 1.1. Priors and Hyperparameters

| component | model | prior | param_a | param_b |
| --- | --- | --- | --- | --- |
| age | RW2 | log-Gamma | 1 | 0.00005 |
| period | RW1 | log-Gamma | 1 | 0.00005 |
| cohort | RW2 | log-Gamma | 1 | 0.00005 |
| overdispersion | IID | log-Gamma | 1 | 0.00500 |

## 1.2. Posterior Predictive Coverage

| target | coverage_95 |
| --- | --- |
| ASR (in-sample) | 0 |
| Age-specific counts (in-sample) | 100 |

## 1.3. Residuals – Overall Summary

| stat | value |
| --- | --- |
| mean_resid | -0.008509932 |
| sd_resid | 0.671484825 |
| prop_abs_resid_gt_2 | 0.000000000 |
| acf_lag1 | 0.180602844 |

## 1.4. Residuals – By Year

| Time | mean_pearson | sd_pearson |
| --- | --- | --- |
| 1,990 | 0.238082499 | 0.6902482 |
| 1,991 | 0.312009942 | 0.6972135 |
| 1,992 | 0.206179841 | 0.8068635 |
| 1,993 | 0.166418503 | 0.8200903 |
| 1,994 | 0.095721915 | 0.8105302 |
| 1,995 | 0.106872044 | 0.7962173 |
| 1,996 | 0.139397398 | 0.7910671 |
| 1,997 | 0.089670698 | 0.7140683 |
| 1,998 | 0.121375226 | 0.5761301 |
| 1,999 | 0.108538270 | 0.4806480 |
| 2,000 | 0.247740983 | 0.3431009 |
| 2,001 | 0.106688018 | 0.2727016 |
| 2,002 | 0.039697303 | 0.1608449 |
| 2,003 | 0.115661103 | 0.6318586 |
| 2,004 | 0.002720643 | 0.6445560 |
| 2,005 | -0.264500270 | 0.4633866 |
| 2,006 | -0.550685041 | 0.7061376 |
| 2,007 | -0.595252513 | 0.9721695 |
| 2,008 | -0.436334106 | 0.6399859 |
| 2,009 | -0.165998592 | 0.7073945 |
| 2,010 | -0.016075308 | 0.9044806 |
| 2,011 | -0.171349398 | 0.9098204 |
| 2,012 | -0.406846237 | 0.9803453 |
| 2,013 | -0.419599527 | 0.8985733 |
| 2,014 | -0.122214530 | 0.4407295 |
| 2,015 | -0.151088975 | 0.3324049 |
| 2,016 | -0.147511715 | 0.3547903 |
| 2,017 | 0.028835427 | 0.3648946 |
| 2,018 | 0.205028674 | 0.5359272 |
| 2,019 | 0.267519873 | 0.5526716 |
| 2,020 | 0.237134731 | 0.6132263 |
| 2,021 | 0.339845285 | 0.7394835 |
