# Supplementary material for: Nationwide Trends and Projections of Early Onset Gastrointestinal Cancers in China
Source: Cancers (Basel). 2025 Sep 9;17(18):2954. doi: 10.3390/cancers17182954 (PMC12468148; doi:10.3390/cancers17182954)
Supplement: Supplementary file 1 [file cancers-17-02954-s001.zip › Supplementary File S1.zip!/Deaths-BAPC_params_output_selected/cause_Liver_cancer/China_Liver_cancer_Both_Deaths_BAPC_parameters_and_ppc.docx]

# 1. Methods (BAPC specification)

BAPC with Poisson likelihood (log link) and population offset. Age/cohort: RW2; period: RW1; IID over-dispersion included. Precision priors: log-Gamma(1, 5e-5) for age/period/cohort; log-Gamma(1, 5e-3) for over-dispersion. Intercept: vague Gaussian prior (package default). Training: 1990–2021; projections to 2040 with retrospective prediction enabled. Age-standardization: WHO 2000–2025 weights.

## 1.1. Priors and Hyperparameters

| component | model | prior | param_a | param_b |
| --- | --- | --- | --- | --- |
| age | RW2 | log-Gamma | 1 | 0.00005 |
| period | RW1 | log-Gamma | 1 | 0.00005 |
| cohort | RW2 | log-Gamma | 1 | 0.00005 |
| overdispersion | IID | log-Gamma | 1 | 0.00500 |

## 1.2. Posterior Predictive Coverage

| target | coverage_95 |
| --- | --- |
| ASR (in-sample) | 0 |
| Age-specific counts (in-sample) | 100 |

## 1.3. Residuals – Overall Summary

| stat | value |
| --- | --- |
| mean_resid | -0.023422471 |
| sd_resid | 0.450448925 |
| prop_abs_resid_gt_2 | 0.004464286 |
| acf_lag1 | 0.000537492 |

## 1.4. Residuals – By Year

| Time | mean_pearson | sd_pearson |
| --- | --- | --- |
| 1,990 | -0.126834268 | 0.2995390 |
| 1,991 | -0.152262269 | 0.3096925 |
| 1,992 | -0.093461769 | 0.2790174 |
| 1,993 | -0.087422170 | 0.2870497 |
| 1,994 | -0.050697756 | 0.3042105 |
| 1,995 | 0.015535001 | 0.4719788 |
| 1,996 | 0.032199605 | 0.5067964 |
| 1,997 | 0.032785701 | 0.4579445 |
| 1,998 | 0.070959706 | 0.3763262 |
| 1,999 | 0.094437305 | 0.3489140 |
| 2,000 | 0.087653219 | 0.3324257 |
| 2,001 | 0.148834743 | 0.3581495 |
| 2,002 | 0.145255703 | 0.3686079 |
| 2,003 | 0.045062311 | 0.5289677 |
| 2,004 | 0.039704001 | 0.5353420 |
| 2,005 | -0.007045012 | 0.5453642 |
| 2,006 | -0.094438973 | 0.6276198 |
| 2,007 | -0.109281706 | 0.6096034 |
| 2,008 | -0.038887514 | 0.4517471 |
| 2,009 | 0.002625295 | 0.3183313 |
| 2,010 | 0.024690087 | 0.3197446 |
| 2,011 | -0.079935384 | 0.3670225 |
| 2,012 | -0.165114889 | 0.5136424 |
| 2,013 | -0.125869077 | 0.6225540 |
| 2,014 | -0.125859316 | 0.7559736 |
| 2,015 | -0.200014259 | 0.8452700 |
| 2,016 | -0.219650981 | 0.7808768 |
| 2,017 | -0.087191124 | 0.4355303 |
| 2,018 | -0.033161869 | 0.3349933 |
| 2,019 | 0.040158842 | 0.2886013 |
| 2,020 | 0.111253881 | 0.3236539 |
| 2,021 | 0.156453849 | 0.4189630 |
