# Supplementary material for: Nationwide Trends and Projections of Early Onset Gastrointestinal Cancers in China
Source: Cancers (Basel). 2025 Sep 9;17(18):2954. doi: 10.3390/cancers17182954 (PMC12468148; doi:10.3390/cancers17182954)
Supplement: Supplementary file 1 [file cancers-17-02954-s001.zip › Supplementary File S1.zip!/Deaths-BAPC_params_output_selected/cause_Esophageal_cancer/China_Esophageal_cancer_Both_Deaths_BAPC_parameters_and_ppc.docx]

# 1. Methods (BAPC specification)

BAPC with Poisson likelihood (log link) and population offset. Age/cohort: RW2; period: RW1; IID over-dispersion included. Precision priors: log-Gamma(1, 5e-5) for age/period/cohort; log-Gamma(1, 5e-3) for over-dispersion. Intercept: vague Gaussian prior (package default). Training: 1990–2021; projections to 2040 with retrospective prediction enabled. Age-standardization: WHO 2000–2025 weights.

## 1.1. Priors and Hyperparameters

| component | model | prior | param_a | param_b |
| --- | --- | --- | --- | --- |
| age | RW2 | log-Gamma | 1 | 0.00005 |
| period | RW1 | log-Gamma | 1 | 0.00005 |
| cohort | RW2 | log-Gamma | 1 | 0.00005 |
| overdispersion | IID | log-Gamma | 1 | 0.00500 |

## 1.2. Posterior Predictive Coverage

| target | coverage_95 |
| --- | --- |
| ASR (in-sample) | 0 |
| Age-specific counts (in-sample) | 100 |

## 1.3. Residuals – Overall Summary

| stat | value |
| --- | --- |
| mean_resid | -0.008853159 |
| sd_resid | 0.389096735 |
| prop_abs_resid_gt_2 | 0.000000000 |
| acf_lag1 | 0.221194999 |

## 1.4. Residuals – By Year

| Time | mean_pearson | sd_pearson |
| --- | --- | --- |
| 1,990 | -0.280489476 | 0.5107436 |
| 1,991 | -0.187738721 | 0.4691167 |
| 1,992 | -0.154320970 | 0.4486875 |
| 1,993 | -0.126398582 | 0.4432347 |
| 1,994 | -0.098064867 | 0.4230203 |
| 1,995 | -0.043330661 | 0.4110234 |
| 1,996 | -0.012671454 | 0.3522085 |
| 1,997 | 0.017387476 | 0.3022501 |
| 1,998 | 0.048722689 | 0.2784199 |
| 1,999 | 0.070323202 | 0.2531300 |
| 2,000 | 0.176502704 | 0.3119384 |
| 2,001 | 0.161467810 | 0.3397020 |
| 2,002 | 0.119306655 | 0.2914584 |
| 2,003 | 0.225512206 | 0.4431492 |
| 2,004 | 0.264091336 | 0.4064264 |
| 2,005 | 0.092333193 | 0.2228792 |
| 2,006 | -0.156938830 | 0.3214518 |
| 2,007 | -0.166201918 | 0.4363509 |
| 2,008 | -0.055421782 | 0.1476207 |
| 2,009 | 0.170200020 | 0.4580516 |
| 2,010 | 0.228687700 | 0.5851923 |
| 2,011 | 0.049899749 | 0.5839578 |
| 2,012 | -0.162957568 | 0.7367519 |
| 2,013 | -0.148387917 | 0.6905990 |
| 2,014 | -0.006091560 | 0.4015283 |
| 2,015 | -0.097371534 | 0.3421287 |
| 2,016 | -0.119051328 | 0.3272821 |
| 2,017 | -0.058121427 | 0.1788757 |
| 2,018 | 0.025874571 | 0.1751024 |
| 2,019 | -0.013158292 | 0.1512064 |
| 2,020 | -0.037939847 | 0.1760927 |
| 2,021 | -0.008953648 | 0.2337329 |
