# Supplementary material for: Nationwide Trends and Projections of Early Onset Gastrointestinal Cancers in China
Source: Cancers (Basel). 2025 Sep 9;17(18):2954. doi: 10.3390/cancers17182954 (PMC12468148; doi:10.3390/cancers17182954)
Supplement: Supplementary file 1 [file cancers-17-02954-s001.zip › Supplementary File S1.zip!/incidence-BAPC_params_output_selected/cause_Stomach_cancer/China_Stomach_cancer_Both_Incidence_BAPC_parameters_and_ppc.docx]

# 1. Methods (BAPC specification)

BAPC with Poisson likelihood (log link) and population offset. Age/cohort: RW2; period: RW1; IID over-dispersion included. Precision priors: log-Gamma(1, 5e-5) for age/period/cohort; log-Gamma(1, 5e-3) for over-dispersion. Intercept: vague Gaussian prior (package default). Training: 1990–2021; projections to 2040 with retrospective prediction enabled. Age-standardization: WHO 2000–2025 weights.

## 1.1. Priors and Hyperparameters

| component | model | prior | param_a | param_b |
| --- | --- | --- | --- | --- |
| age | RW2 | log-Gamma | 1 | 0.00005 |
| period | RW1 | log-Gamma | 1 | 0.00005 |
| cohort | RW2 | log-Gamma | 1 | 0.00005 |
| overdispersion | IID | log-Gamma | 1 | 0.00500 |

## 1.2. Posterior Predictive Coverage

| target | coverage_95 |
| --- | --- |
| ASR (in-sample) | 0 |
| Age-specific counts (in-sample) | 100 |

## 1.3. Residuals – Overall Summary

| stat | value |
| --- | --- |
| mean_resid | -0.0172857 |
| sd_resid | 0.3699356 |
| prop_abs_resid_gt_2 | 0.0000000 |
| acf_lag1 | 0.8785737 |

## 1.4. Residuals – By Year

| Time | mean_pearson | sd_pearson |
| --- | --- | --- |
| 1,990 | 0.088938273 | 0.57316635 |
| 1,991 | 0.101650896 | 0.57266099 |
| 1,992 | 0.112537472 | 0.55494955 |
| 1,993 | 0.107114731 | 0.53524777 |
| 1,994 | 0.093799789 | 0.47467832 |
| 1,995 | 0.107534704 | 0.42758983 |
| 1,996 | 0.076211878 | 0.35895938 |
| 1,997 | 0.028435113 | 0.27795438 |
| 1,998 | 0.006920432 | 0.19251160 |
| 1,999 | -0.003053094 | 0.11957015 |
| 2,000 | 0.023534257 | 0.06046927 |
| 2,001 | -0.027272775 | 0.08842934 |
| 2,002 | -0.052583050 | 0.14592850 |
| 2,003 | -0.014859500 | 0.16713840 |
| 2,004 | 0.022660405 | 0.16059915 |
| 2,005 | -0.054660736 | 0.21056176 |
| 2,006 | -0.215008161 | 0.32256277 |
| 2,007 | -0.230730043 | 0.33450196 |
| 2,008 | -0.159973011 | 0.29427139 |
| 2,009 | -0.048796901 | 0.39304571 |
| 2,010 | -0.026399331 | 0.43765822 |
| 2,011 | -0.104607692 | 0.45927576 |
| 2,012 | -0.228482530 | 0.50749749 |
| 2,013 | -0.211913001 | 0.49105977 |
| 2,014 | -0.113253361 | 0.40051667 |
| 2,015 | -0.149312519 | 0.37952663 |
| 2,016 | -0.119869486 | 0.36401076 |
| 2,017 | -0.016054513 | 0.34298627 |
| 2,018 | 0.061175557 | 0.36286463 |
| 2,019 | 0.094173570 | 0.37754929 |
| 2,020 | 0.125591169 | 0.40626429 |
| 2,021 | 0.173409121 | 0.45410746 |
