# Supplementary material for: Nationwide Trends and Projections of Early Onset Gastrointestinal Cancers in China
Source: Cancers (Basel). 2025 Sep 9;17(18):2954. doi: 10.3390/cancers17182954 (PMC12468148; doi:10.3390/cancers17182954)
Supplement: Supplementary file 1 [file cancers-17-02954-s001.zip › Supplementary File S1.zip!/incidence-BAPC_params_output_selected/cause_Colon_and_rectum_cancer/China_Colon_and_rectum_cancer_Both_Incidence_BAPC_parameters_and_ppc.docx]

# 1. Methods (BAPC specification)

BAPC with Poisson likelihood (log link) and population offset. Age/cohort: RW2; period: RW1; IID over-dispersion included. Precision priors: log-Gamma(1, 5e-5) for age/period/cohort; log-Gamma(1, 5e-3) for over-dispersion. Intercept: vague Gaussian prior (package default). Training: 1990–2021; projections to 2040 with retrospective prediction enabled. Age-standardization: WHO 2000–2025 weights.

## 1.1. Priors and Hyperparameters

| component | model | prior | param_a | param_b |
| --- | --- | --- | --- | --- |
| age | RW2 | log-Gamma | 1 | 0.00005 |
| period | RW1 | log-Gamma | 1 | 0.00005 |
| cohort | RW2 | log-Gamma | 1 | 0.00005 |
| overdispersion | IID | log-Gamma | 1 | 0.00500 |

## 1.2. Posterior Predictive Coverage

| target | coverage_95 |
| --- | --- |
| ASR (in-sample) | 0 |
| Age-specific counts (in-sample) | 100 |

## 1.3. Residuals – Overall Summary

| stat | value |
| --- | --- |
| mean_resid | -0.01711988 |
| sd_resid | 0.32150552 |
| prop_abs_resid_gt_2 | 0.00000000 |
| acf_lag1 | 0.89707969 |

## 1.4. Residuals – By Year

| Time | mean_pearson | sd_pearson |
| --- | --- | --- |
| 1,990 | 0.072546753 | 0.44640814 |
| 1,991 | 0.074962240 | 0.44843894 |
| 1,992 | 0.066755311 | 0.45415469 |
| 1,993 | 0.066809659 | 0.45193860 |
| 1,994 | 0.060490660 | 0.41023651 |
| 1,995 | 0.064570548 | 0.39422061 |
| 1,996 | 0.064300595 | 0.36348952 |
| 1,997 | 0.053151866 | 0.33097991 |
| 1,998 | 0.051401559 | 0.27911046 |
| 1,999 | 0.045809859 | 0.24165377 |
| 2,000 | 0.063425031 | 0.19343364 |
| 2,001 | 0.010634060 | 0.12054022 |
| 2,002 | -0.042968486 | 0.07494675 |
| 2,003 | -0.043769355 | 0.16742317 |
| 2,004 | -0.048648025 | 0.20992338 |
| 2,005 | -0.106234451 | 0.26561585 |
| 2,006 | -0.213231284 | 0.37451191 |
| 2,007 | -0.224611451 | 0.38450586 |
| 2,008 | -0.187602569 | 0.36286578 |
| 2,009 | -0.119260423 | 0.40576590 |
| 2,010 | -0.093009380 | 0.41577287 |
| 2,011 | -0.114142793 | 0.38562455 |
| 2,012 | -0.154935981 | 0.38078699 |
| 2,013 | -0.128525069 | 0.34353222 |
| 2,014 | -0.028990374 | 0.25020521 |
| 2,015 | -0.045791563 | 0.22537483 |
| 2,016 | -0.056322040 | 0.24519886 |
| 2,017 | 0.007570758 | 0.24818728 |
| 2,018 | 0.064345512 | 0.27215714 |
| 2,019 | 0.082277687 | 0.29296300 |
| 2,020 | 0.087959794 | 0.31028715 |
| 2,021 | 0.123195127 | 0.34615189 |
