# Supplementary material for: Nationwide Trends and Projections of Early Onset Gastrointestinal Cancers in China
Source: Cancers (Basel). 2025 Sep 9;17(18):2954. doi: 10.3390/cancers17182954 (PMC12468148; doi:10.3390/cancers17182954)
Supplement: Supplementary file 1 [file cancers-17-02954-s001.zip › Supplementary File S1.zip!/incidence-BAPC_params_output_selected/cause_Gallbladder_and_biliary_tract_cancer/China_Gallbladder_and_biliary_tract_cancer_Both_Incidence_BAPC_parameters_and_ppc.docx]

# 1. Methods (BAPC specification)

BAPC with Poisson likelihood (log link) and population offset. Age/cohort: RW2; period: RW1; IID over-dispersion included. Precision priors: log-Gamma(1, 5e-5) for age/period/cohort; log-Gamma(1, 5e-3) for over-dispersion. Intercept: vague Gaussian prior (package default). Training: 1990–2021; projections to 2040 with retrospective prediction enabled. Age-standardization: WHO 2000–2025 weights.

## 1.1. Priors and Hyperparameters

| component | model | prior | param_a | param_b |
| --- | --- | --- | --- | --- |
| age | RW2 | log-Gamma | 1 | 0.00005 |
| period | RW1 | log-Gamma | 1 | 0.00005 |
| cohort | RW2 | log-Gamma | 1 | 0.00005 |
| overdispersion | IID | log-Gamma | 1 | 0.00500 |

## 1.2. Posterior Predictive Coverage

| target | coverage_95 |
| --- | --- |
| ASR (in-sample) | 0 |
| Age-specific counts (in-sample) | 100 |

## 1.3. Residuals – Overall Summary

| stat | value |
| --- | --- |
| mean_resid | -0.02851488 |
| sd_resid | 0.50912791 |
| prop_abs_resid_gt_2 | 0.00000000 |
| acf_lag1 | 0.83204895 |

## 1.4. Residuals – By Year

| Time | mean_pearson | sd_pearson |
| --- | --- | --- |
| 1,990 | 0.344447871 | 0.5158243 |
| 1,991 | 0.305002114 | 0.5022564 |
| 1,992 | 0.170235142 | 0.4563304 |
| 1,993 | 0.115291757 | 0.4665694 |
| 1,994 | 0.095073685 | 0.4956793 |
| 1,995 | 0.056934733 | 0.5233131 |
| 1,996 | -0.007161678 | 0.5518980 |
| 1,997 | -0.087987472 | 0.5442234 |
| 1,998 | -0.143091196 | 0.4133772 |
| 1,999 | -0.171197966 | 0.2820733 |
| 2,000 | -0.019476449 | 0.2599963 |
| 2,001 | -0.217176714 | 0.2693186 |
| 2,002 | -0.147315305 | 0.3475328 |
| 2,003 | 0.094314279 | 0.7343651 |
| 2,004 | 0.079013578 | 0.7480460 |
| 2,005 | -0.035065653 | 0.4960032 |
| 2,006 | -0.204691247 | 0.4030711 |
| 2,007 | -0.229926548 | 0.6665830 |
| 2,008 | -0.230991070 | 0.4516812 |
| 2,009 | -0.087491612 | 0.3918759 |
| 2,010 | -0.049813114 | 0.5172802 |
| 2,011 | -0.101263036 | 0.5763789 |
| 2,012 | -0.273407086 | 0.6760695 |
| 2,013 | -0.233155747 | 0.6077901 |
| 2,014 | -0.006776598 | 0.4635354 |
| 2,015 | -0.097602069 | 0.5132724 |
| 2,016 | -0.149205492 | 0.4932502 |
| 2,017 | -0.044187597 | 0.5128969 |
| 2,018 | 0.123571885 | 0.6591819 |
| 2,019 | 0.145702888 | 0.6089365 |
| 2,020 | 0.041407633 | 0.5455865 |
| 2,021 | 0.053511786 | 0.5941481 |
