# Supplementary material for: Nationwide Trends and Projections of Early Onset Gastrointestinal Cancers in China
Source: Cancers (Basel). 2025 Sep 9;17(18):2954. doi: 10.3390/cancers17182954 (PMC12468148; doi:10.3390/cancers17182954)
Supplement: Supplementary file 1 [file cancers-17-02954-s001.zip › Supplementary File S1.zip!/incidence-BAPC_params_output_selected/cause_Pancreatic_cancer/China_Pancreatic_cancer_Both_Incidence_BAPC_parameters_and_ppc.docx]

# 1. Methods (BAPC specification)

BAPC with Poisson likelihood (log link) and population offset. Age/cohort: RW2; period: RW1; IID over-dispersion included. Precision priors: log-Gamma(1, 5e-5) for age/period/cohort; log-Gamma(1, 5e-3) for over-dispersion. Intercept: vague Gaussian prior (package default). Training: 1990–2021; projections to 2040 with retrospective prediction enabled. Age-standardization: WHO 2000–2025 weights.

## 1.1. Priors and Hyperparameters

| component | model | prior | param_a | param_b |
| --- | --- | --- | --- | --- |
| age | RW2 | log-Gamma | 1 | 0.00005 |
| period | RW1 | log-Gamma | 1 | 0.00005 |
| cohort | RW2 | log-Gamma | 1 | 0.00005 |
| overdispersion | IID | log-Gamma | 1 | 0.00500 |

## 1.2. Posterior Predictive Coverage

| target | coverage_95 |
| --- | --- |
| ASR (in-sample) | 0 |
| Age-specific counts (in-sample) | 100 |

## 1.3. Residuals – Overall Summary

| stat | value |
| --- | --- |
| mean_resid | -0.008204886 |
| sd_resid | 0.672957632 |
| prop_abs_resid_gt_2 | 0.000000000 |
| acf_lag1 | 0.829637033 |

## 1.4. Residuals – By Year

| Time | mean_pearson | sd_pearson |
| --- | --- | --- |
| 1,990 | 0.270688687 | 0.7251648 |
| 1,991 | 0.345106767 | 0.7359852 |
| 1,992 | 0.204204369 | 0.7969580 |
| 1,993 | 0.192448611 | 0.8515225 |
| 1,994 | 0.122775236 | 0.8363564 |
| 1,995 | 0.127001157 | 0.8186675 |
| 1,996 | 0.134671564 | 0.7769026 |
| 1,997 | 0.095023304 | 0.7100510 |
| 1,998 | 0.106105039 | 0.5602870 |
| 1,999 | 0.115294483 | 0.4751652 |
| 2,000 | 0.237160392 | 0.3284129 |
| 2,001 | 0.089612265 | 0.2484792 |
| 2,002 | 0.031172331 | 0.1507896 |
| 2,003 | 0.089300172 | 0.6221224 |
| 2,004 | 0.007136296 | 0.6160459 |
| 2,005 | -0.280991744 | 0.4695145 |
| 2,006 | -0.575420697 | 0.7359585 |
| 2,007 | -0.590872188 | 0.9663534 |
| 2,008 | -0.438308263 | 0.6382609 |
| 2,009 | -0.174837996 | 0.7139305 |
| 2,010 | -0.035112188 | 0.9091188 |
| 2,011 | -0.146811757 | 0.8770073 |
| 2,012 | -0.403243869 | 0.9703001 |
| 2,013 | -0.400612383 | 0.8759441 |
| 2,014 | -0.116993245 | 0.4567843 |
| 2,015 | -0.159504547 | 0.3369772 |
| 2,016 | -0.162718946 | 0.3479628 |
| 2,017 | 0.009647406 | 0.3654312 |
| 2,018 | 0.199565741 | 0.5412453 |
| 2,019 | 0.267848465 | 0.5207820 |
| 2,020 | 0.259876427 | 0.6074242 |
| 2,021 | 0.318232773 | 0.7428148 |
