# Supplementary material for: Nationwide Trends and Projections of Early Onset Gastrointestinal Cancers in China
Source: Cancers (Basel). 2025 Sep 9;17(18):2954. doi: 10.3390/cancers17182954 (PMC12468148; doi:10.3390/cancers17182954)
Supplement: Supplementary file 1 [file cancers-17-02954-s001.zip › Supplementary File S1.zip!/incidence-BAPC_params_output_selected/cause_Liver_cancer/China_Liver_cancer_Both_Incidence_BAPC_parameters_and_ppc.docx]

# 1. Methods (BAPC specification)

BAPC with Poisson likelihood (log link) and population offset. Age/cohort: RW2; period: RW1; IID over-dispersion included. Precision priors: log-Gamma(1, 5e-5) for age/period/cohort; log-Gamma(1, 5e-3) for over-dispersion. Intercept: vague Gaussian prior (package default). Training: 1990–2021; projections to 2040 with retrospective prediction enabled. Age-standardization: WHO 2000–2025 weights.

## 1.1. Priors and Hyperparameters

| component | model | prior | param_a | param_b |
| --- | --- | --- | --- | --- |
| age | RW2 | log-Gamma | 1 | 0.00005 |
| period | RW1 | log-Gamma | 1 | 0.00005 |
| cohort | RW2 | log-Gamma | 1 | 0.00005 |
| overdispersion | IID | log-Gamma | 1 | 0.00500 |

## 1.2. Posterior Predictive Coverage

| target | coverage_95 |
| --- | --- |
| ASR (in-sample) | 0 |
| Age-specific counts (in-sample) | 100 |

## 1.3. Residuals – Overall Summary

| stat | value |
| --- | --- |
| mean_resid | -0.022023022 |
| sd_resid | 0.505303635 |
| prop_abs_resid_gt_2 | 0.008928571 |
| acf_lag1 | 0.916886474 |

## 1.4. Residuals – By Year

| Time | mean_pearson | sd_pearson |
| --- | --- | --- |
| 1,990 | -0.213806535 | 0.4183514 |
| 1,991 | -0.132880830 | 0.3397442 |
| 1,992 | -0.084652686 | 0.3560400 |
| 1,993 | -0.053793548 | 0.4245095 |
| 1,994 | -0.024363139 | 0.4871989 |
| 1,995 | -0.002006767 | 0.5273529 |
| 1,996 | 0.019814623 | 0.5263149 |
| 1,997 | 0.044810787 | 0.4812693 |
| 1,998 | 0.072024893 | 0.4211115 |
| 1,999 | 0.096472043 | 0.3762289 |
| 2,000 | 0.131050900 | 0.3524947 |
| 2,001 | 0.147608098 | 0.3294644 |
| 2,002 | 0.125394503 | 0.3152689 |
| 2,003 | 0.074201273 | 0.4325736 |
| 2,004 | -0.006403830 | 0.6307773 |
| 2,005 | -0.033569373 | 0.7235271 |
| 2,006 | -0.007354763 | 0.6581316 |
| 2,007 | -0.005769694 | 0.5453349 |
| 2,008 | -0.011568193 | 0.4492299 |
| 2,009 | -0.014132683 | 0.3907053 |
| 2,010 | -0.010267314 | 0.3821294 |
| 2,011 | -0.032459755 | 0.3974685 |
| 2,012 | -0.092968974 | 0.5236860 |
| 2,013 | -0.170329218 | 0.7461934 |
| 2,014 | -0.232918163 | 0.9333009 |
| 2,015 | -0.252088700 | 0.9624042 |
| 2,016 | -0.212439663 | 0.8127063 |
| 2,017 | -0.130868161 | 0.5910826 |
| 2,018 | -0.042680436 | 0.3946105 |
| 2,019 | 0.042790797 | 0.3191853 |
| 2,020 | 0.129556725 | 0.3615620 |
| 2,021 | 0.178861066 | 0.4654917 |
