# Supplementary material for: Nationwide Trends and Projections of Early Onset Gastrointestinal Cancers in China
Source: Cancers (Basel). 2025 Sep 9;17(18):2954. doi: 10.3390/cancers17182954 (PMC12468148; doi:10.3390/cancers17182954)
Supplement: Supplementary file 1 [file cancers-17-02954-s001.zip › Supplementary File S1.zip!/incidence-BAPC_params_output_selected/cause_Esophageal_cancer/China_Esophageal_cancer_Both_Incidence_BAPC_parameters_and_ppc.docx]

# 1. Methods (BAPC specification)

BAPC with Poisson likelihood (log link) and population offset. Age/cohort: RW2; period: RW1; IID over-dispersion included. Precision priors: log-Gamma(1, 5e-5) for age/period/cohort; log-Gamma(1, 5e-3) for over-dispersion. Intercept: vague Gaussian prior (package default). Training: 1990–2021; projections to 2040 with retrospective prediction enabled. Age-standardization: WHO 2000–2025 weights.

## 1.1. Priors and Hyperparameters

| component | model | prior | param_a | param_b |
| --- | --- | --- | --- | --- |
| age | RW2 | log-Gamma | 1 | 0.00005 |
| period | RW1 | log-Gamma | 1 | 0.00005 |
| cohort | RW2 | log-Gamma | 1 | 0.00005 |
| overdispersion | IID | log-Gamma | 1 | 0.00500 |

## 1.2. Posterior Predictive Coverage

| target | coverage_95 |
| --- | --- |
| ASR (in-sample) | 0 |
| Age-specific counts (in-sample) | 100 |

## 1.3. Residuals – Overall Summary

| stat | value |
| --- | --- |
| mean_resid | 0.04971383 |
| sd_resid | 0.35408789 |
| prop_abs_resid_gt_2 | 0.00000000 |
| acf_lag1 | 0.83085435 |

## 1.4. Residuals – By Year

| Time | mean_pearson | sd_pearson |
| --- | --- | --- |
| 1,990 | -0.164692759 | 0.4955033 |
| 1,991 | -0.095125529 | 0.4601393 |
| 1,992 | -0.068669530 | 0.4370769 |
| 1,993 | -0.031790300 | 0.4389568 |
| 1,994 | -0.017556841 | 0.4007880 |
| 1,995 | 0.029690425 | 0.3818329 |
| 1,996 | 0.052680565 | 0.3427435 |
| 1,997 | 0.072050064 | 0.2821711 |
| 1,998 | 0.101640166 | 0.2596473 |
| 1,999 | 0.123080264 | 0.2460543 |
| 2,000 | 0.199289302 | 0.2986039 |
| 2,001 | 0.192942379 | 0.3239416 |
| 2,002 | 0.145553525 | 0.2848586 |
| 2,003 | 0.228980685 | 0.3873690 |
| 2,004 | 0.280755586 | 0.3719980 |
| 2,005 | 0.128993088 | 0.2410829 |
| 2,006 | -0.086093149 | 0.3502380 |
| 2,007 | -0.094024958 | 0.4276178 |
| 2,008 | 0.016762911 | 0.1787821 |
| 2,009 | 0.195163906 | 0.4080449 |
| 2,010 | 0.244441992 | 0.4933464 |
| 2,011 | 0.103089475 | 0.4874658 |
| 2,012 | -0.069454140 | 0.6182703 |
| 2,013 | -0.047399330 | 0.5690438 |
| 2,014 | 0.076763583 | 0.3220417 |
| 2,015 | -0.023301479 | 0.2610511 |
| 2,016 | -0.063175339 | 0.2610800 |
| 2,017 | 0.006904698 | 0.1625418 |
| 2,018 | 0.065648878 | 0.1738447 |
| 2,019 | 0.039414385 | 0.1611008 |
| 2,020 | 0.012772908 | 0.1895524 |
| 2,021 | 0.035507137 | 0.2390843 |
